# Construction of Nomograms for Predicting Lung and Bone Metastases in Patients with Intrahepatic Cholangiocarcinoma and Identification of Patients Who Can Benefit from Chemotherapy

**DOI:** 10.1155/2020/8889571

**Published:** 2020-12-02

**Authors:** Ning Shi, Yiping Zou, Yuanpeng Zhang, Hongwei Han, Zhihong Chen, Shiye Ruan, Liang Jin, Zuyi Ma, Zhenrong Chen, Qi Lou, Haosheng Jin

**Affiliations:** ^1^Department of General Surgery, Guangdong Provincial People's Hospital, Guangdong Academy of Medical Sciences, Guangzhou, China; ^2^Shantou University Medical College, Shantou, China; ^3^The Second School of Clinical Medicine, Southern Medical University, Guangzhou,China

## Abstract

**Objective:**

The purpose of our study is to build nomograms for predicting the possibility of lung metastasis (LM) and bone metastasis (BM) in patients with intrahepatic cholangiocarcinoma (ICC).

**Methods:**

1527 patients diagnosed with ICC between 2010 and 2016 were collected from the Surveillance, Epidemiology, and End Results (SEER) database. Univariable and multivariable logistic regression analyses were used to recognize the predictors of LM and BM, respectively. Then two nomograms were established. We applied the C-index, calibration plot, receiver-operating characteristic (ROC) curve, and decision curve analysis (DCA) to evaluate the novel nomograms. The maximum values of the Youden indexes from the ROC curves were utilized to select the cutoff points of the nomograms. The Kaplan–Meier survival curves were used to evaluate the effect of chemotherapy in different groups. The bootstrap resampling method was chosen for internal validation.

**Results:**

Five predictors for LM and three predictors for BM were identified, and two nomograms were constructed. The nomograms had high values of C-indexes, reaching 0.821 (95% CI 0.772–0.871) for LM and 0.759 (95% CI 0.700–0.818) for BM. C-indexes of 0.814 for LM and 0.749 for BM were also observed in internal validation. The calibration plots, ROC curves, and DCAs exhibited favorable performances for predicting LM and BM. The cutoff points of total points in nomograms were 108 for LM and 144 for BM, which could distinguish between high-risk and low-risk groups for LM and BM. Chemotherapy is suggested to undergo for patients in high-risk groups.

**Conclusions:**

The nomograms could assess the possibility of LM and BM in ICC patients and determine the optimal treatment.

## 1. Introduction

Intrahepatic cholangiocarcinoma (ICC) originates from the epithelial lining of the intrahepatic bile duct, which is the second most universal primary liver cancer [1–3]. Though being a relatively rare tumor, the global morbidity and mortality rates of ICC have increased over the last several decades [4].

ICC is a lethal disease which has an extremely poor prognosis, with a 5-year survival rate ranging from 15–40%. Surgical resection remains the only potential curative treatment. However, only 30–40% of ICC patients are potential to undergo radical surgery, and the postoperative recurrence rate is high, which ranges from 40% to 80% [5–7]. In addition, extrahepatic recurrence is discovered in nearly 40% of postoperative recurrence cases [8]. Compared with hepatocellular carcinoma (HCC), ICC is more invasive and has a higher probability of metastasis [9]. Patients with distant metastasis were always considered to receive systemic therapy (including systemic chemotherapy and targeted therapy) [10]. Therefore, in order to improve prognosis of ICC patients, it is crucial to detect the ICC metastasis early and make proper therapeutic strategies. A retrospective study discovered that the lung was the most common site of ICC distant metastases, followed by the bone [11]. In order to determine the possibility of resection for ICC patients, it is recommended to combine multiphasic CT/MRI with IV contrast of abdomen and chest CT (with or without contrast) [12, 13]. However, it is difficult for the radiologist to find some atypical lung lesions, and identifying the small lung nodules is also hard [14]. Additionally, positron emission tomography-computed tomography (PET-CT) is a valid examination to improve the accuracy for detection of regional lymph node metastasis and unsuspected distant metastasis in patients with cholangiocarcinoma [15]. However, it is not profitable to choose PET-CT as the primary examination for initial diagnosis of all ICC patients due to the expensive price and small proportion of positive patients. Therefore, identifying patients in high risk of lung metastasis (LM) and bone metastasis (BM) is important to determine the optimal treatment guidance. In other words, it is necessary to distinguish patients with high risk of LM and BM from others.

Up to now, a model that preliminarily finds out whether patients with ICC are at high risk of LM and BM does not exist yet. A nomogram is a kind of dependable graphical math model that is used to exactly predict a specific end event in combination with the related risk factors of tumor development [16, 17]. Therefore, the original purpose of our study is to recognize independent risk factors promoting LM and BM for ICC patients and to build nomograms for predicting the risk of LM and BM. By using these nomograms, clinicians can stratify patients with higher risk of LM and BM and determine the optimal therapeutic strategy in clinical application.

## 2. Materials and Methods

### 2.1. Materials

We analyzed the Surveillance, Epidemiology, and End Results (SEER) database, a National *Cancer* Institute- (NCI-) initiated registry of cancer incidence and survival rates in the United States from 1975 to 2016. We got the permission from SEER to access the research data in November 2018 for analysis (Username: 14376-Nov2018). We used SEER∗Stat software version 8.3.6 to extract patients diagnosed with ICC between 2010 and 2016 from the SEER database. The SEER database is publicly available, and all patient data have been unlabeled, so informed consent from the institutional review board is not required for this research.

The retrospective population study cohort included the following patients with international tumor classification, histology code (ICD-O-3): 8160, and the ICD site code: C22.1. The exclusion criteria of this study were listed as follows: [1] type of reporting source: autopsy only or death certificate only; [2] patients who lacked of information about the location of distant metastasis and clinicopathological features; [3] patients with second primary cancer; [4] patients younger than 18 years of age; and [5] patients diagnosed without positive histology. The analyzed clinicopathological features included age at diagnosis, gender, race, histological grade, and marital status at diagnosis, 7th American Joint Committee on *Cancer* (AJCC) *T* stage and 7th AJCC N stage.

### 2.2. Statistical Analysis

Patient characteristics were summarized as the *n* (%) for categorical variables. Univariate logistic regression analysis was applied to select the associated risk predictors of LM and BM. Multivariate logistic regression analysis was further utilized to define the independent risk predictors of LM and BM. We also calculated the odds ratios (OR) and the 95% confidence intervals (CI) of them to compare the risk levels of the predictors.

Based on the independent predictors of LM and BM searched from the multivariate logistic analysis, two nomograms were constructed. To further evaluate the benefits of the novel models, *C*-indexes in addition with receiver operating characteristic (ROC) curves were used to assess the discrimination. The cutoff values of the total scores from the nomograms were calculated according to the maximum values of the Youden index for the ROC curves. Sensitivity and specificity, positive predictive value, and negative predictive value were also displayed. Low-risk groups and high-risk groups of patients were divided by these values. In order to estimate the predictive accuracy and bias, calibration curves with 1,000 bootstrap resampling were conducted. The decision curve analyses (DCA) were utilized to evaluate the clinical use of nomograms. For internal validation of the novel nomograms, we used a bootstrap resampling method with 500 repetition. The Kaplan–Meier survival curves of the high-risk and low-risk groups for LM and BM with the Log-Rank test were plotted to compare their overall survival. Furthermore, the Kaplan–Meier survival curves of patients with or without chemotherapy in the high-risk and low-risk groups were plotted, respectively.

The statistical analysis of this study was performed using *R* software, version 3.6.1 (http://www.r-project.org/). The *p* values <0.1 for univariate logistic regression analysis and <0.05 for other analyses were considered statistically significant.

## 3. Results

### 3.1. Patient Baseline Characteristics and Logistic Regression Analyses

**Figure 1:**
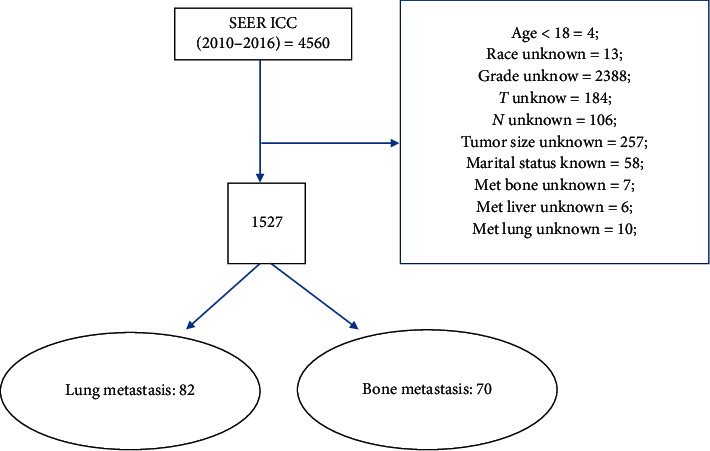
Flow diagram for selecting ICC patients from the SEER database. ICC: intrahepatic carcinoma; SEER: Surveillance, Epidemiology, and End Results.

After screening with the exclusion criteria of our study, a total of 1527 patients with ICC were involved. Among them, 82 people had LM and 70 had BM. The detailed process of inclusion and exclusion is shown in Figure 1.  Table 1 summarizes the data of patients with clinical characteristics and demographics. There are four columns in this table which correspond to patients with or without LM and BM.

To filter risk factors of LM and BM for ICC patients, the univariate logistic regression analysis was performed. There were six clinicopathological variables related to LM and five clinicopathological variables related to BM. The multivariate logistic regression analyses were used to analyze these variables. After gradually removing variables, five significant variables were actually picked out for LM: gender (male: OR 2.253, 95% CI 1.365–3.817, *p* = 0.002), histological grade (poor/undifferentiated: OR 1.791, 95% CI 1.094–2.957, *p* = 0.021), *N* stage (*N*1 : OR 1.611, 95% CI 0.978–2.641, *p* = 0.050), tumor size (5–10 cm: OR 3.171, 95% CI 1.565–7.014, *p* = 0.002; >5 cm: OR 8.133, 95% CI 3.867–18.563, *p* < 0.001), and intrahepatic metastasis (Yes : OR 10.431, 95% CI 5.768–18.707, *p* < 0.001). In addition, three significant variables were actually picked out for BM: gender (male: OR 2.870, 95% CI 1.663–5.176, *p* < 0.001), tumor size (5–10 cm: OR 3.601, 95% CI 1.834–7.751, *p* < 0.001; >5 cm: OR 4.551, 95% CI 2.107–10.434, *p* < 0.001), and intrahepatic metastasis (Yes : OR 5.198, 95% CI 2.632–9.817, *p* < 0.001). The results of logistic regression analyses for LM are shown in Table 2, while the results of logistic regression analyses for BM are shown in Table 3.

### 3.2. Nomogram Construction

**Figure 2:**
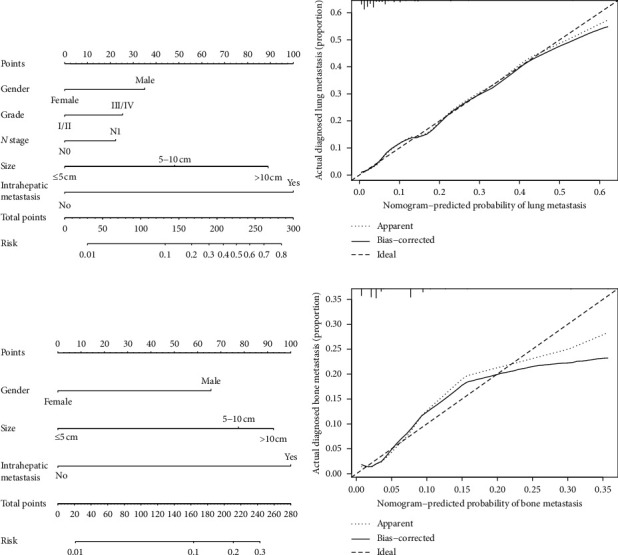
Nomograms for predicting risk of lung and bone metastases in patients with ICC. (a) Nomogram estimated by clinical features for possibility of LM in patients with ICC; (b) calibration curve showing nomogram-predicted LM probabilities compared with the actual LM metastasis; (c) nomogram estimated by clinical features for possibility of BM in patients with ICC; (d) calibration curve showing nomogram-predicted BM probabilities compared with the actual BM metastasis. LM: lung metastasis; BM: bone metastasis; ICC: intrahepatic carcinoma.

Two nomograms were constructed to predict the possibility of LM and BM in patients with ICC based on the predictors searched from multivariate logistic analyses (Figures 2(a) and 2(c)). Intrahepatic metastasis was set as reference because it had the largest absolute value of the coefficient in two nomograms. The scale ranges of the nomograms were from 0 to 100. According to the scales mentioned above, we could obtain score value of each predictor which was marked on its line (LM: female = 0, male = 35, Grade I/II = 0, Grade III/IV = 25, *N*0 = 0, *N*1 = 22, size ≤ 5 cm = 0, size 5–10 cm = 48, size > 10 cm = 89, without intrahepatic metastasis = 0, with intrahepatic metastasis = 100; BM: female = 0, male = 66, size ≤ 5 cm = 0, size 5–10 cm = 78, size > 10 cm = 93, without intrahepatic metastasis = 0, with intrahepatic metastasis = 100). According to these nomograms, the probability of LM and BM could be easily calculated, by summing up the total scores of each predictor as there were parallel lines below the figures whose scales had a linear relationship with each other. This result implied that we could predict the possibilities of LM and BM when a patient's clinicopathologic characteristics were given explicitly.

### 3.3. Apparent Performances of the LM and BM Risk Nomograms

**Figure 3:**
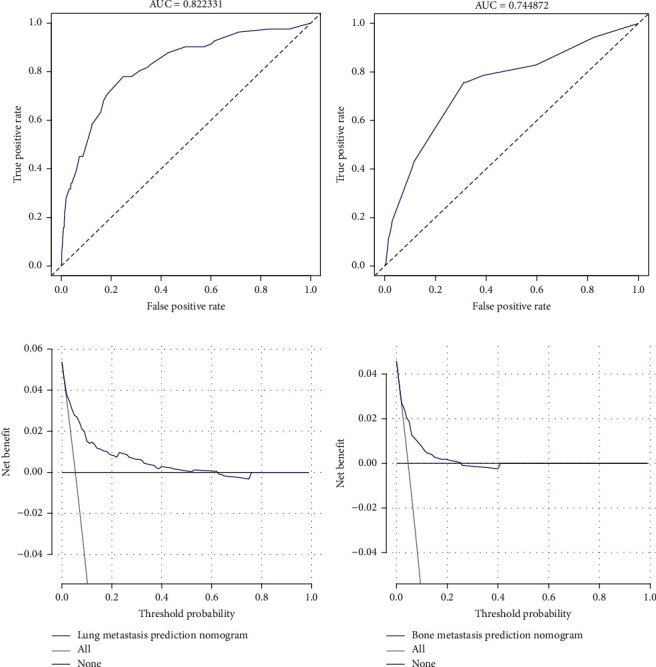
ROCs and DCAs for LM and BM nomograms. (a) The ROC curve for the LM nomogram. (b) The ROC curve for the BM nomogram. (c) DCA for the LM nomogram. (d) DCA for the BM nomogram. ROC: receiver operating characteristic; LM: lung metastasis; BM: bone metastasis; DCA: decision curve analysis.

The nomograms demonstrated good accuracy, with C-indexes of 0.821 (95% CI 0.772–0.871) for LM and 0.769 (95% CI = 0.737–0.801) for BM. We performed the calibration of the LM and BM nomograms internally with 1000 times bootstrap sampling. The calibration curves of the LM and BM risk-predicting nomograms demonstrated good agreement between prediction and observation (Figures 2(b) and 2(d)). Then, the ROC curves of LM and BM nomograms were plotted. The area under curve (AUC) of the LM and BM nomograms were 0.822 and 0.745, respectively (Figures 3(a) and 3(b)). The bootstrap resampling method selecting 500 repetitions was utilized for internal validation of the novel nomograms, and C-indexes of 0.814 for LM and 0.749 for BM were also observed. All of the results mentioned above indicated that two novel nomograms demonstrated good discriminations and fitted well internally. In order to assess predictive models from the perspective of clinical outcomes, the DCAs of two nomograms were presented. Threshold probabilities of 0–0.63 for LM (Figure 3(c)) or 0–0.25 for BM (Figure 3(d)) were the most beneficial for predicting LM and BM with two nomograms. In other words, the nomograms provided additional values relative to the treat-all patients scheme or the treat-none scheme in these threshold probabilities. This result suggested that these two models were extremely useful for clinical determinations.

### 3.4. Performances of the Nomograms in Risk Stratification of Patients

**Figure 4:**
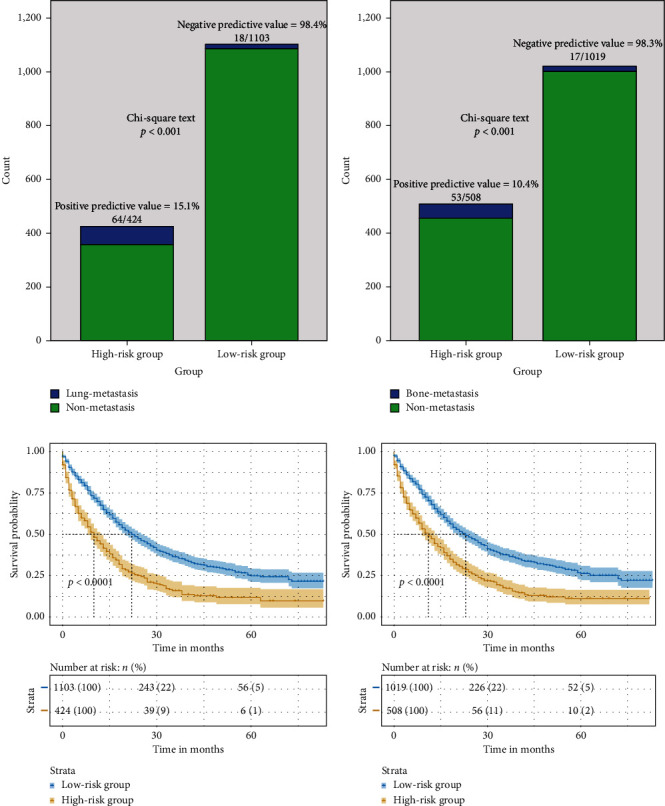
Classification of risk groups for the LM and BM nomograms. (a) Classification of risk groups for the LM nomogram conducted by the maximum value of the Youden index from the ROC curve and their performance in distinguishing LM. (b) Classification of risk groups for the BM nomogram conducted by the maximum value of the Youden index from the ROC curve and their performance in distinguishing BM. (c) Kaplan–Meier curves of OS for ICC patients stratified by high-risk and low-risk groups of LM; (d) Kaplan–Meier curves of OS for ICC patients stratified by high-risk and low-risk groups of BM. LM: lung metastasis; BM: bone metastasis; ROC: receiver operating characteristic; OS: overall survival; ICC: intrahepatic carcinoma.

We obtained the cutoff values of 108 for the LM nomogram and 144 for BM nomogram from the maximum values of the Youden indexes of the ROC curves. All ICC patients were divided into a low-risk group and a high-risk group for LM (total point < 108: low-risk group; total point ≥ 108: high-risk group) and BM (total point < 144: low-risk group; total point ≥ 144: high-risk group), respectively.  Figure 4 shows that high-risk groups had a higher possibility of LM and BM than the low-risk groups (*p* < 0.0001). The optimal cutoff value of LM had a sensitivity of 78.0%, a specificity of 75.1%, a positive predictive value of 15.1%, and a negative predictive value of 98.4%. Similarly, the optimal cutoff value of LM had a sensitivity of 75.7%, a specificity of 68.8%, a positive predictive value of 10.4%, and a negative predictive value of 98.3%. Furthermore, the Kaplan–Meier survival curves of patients in the high-risk groups and low-risk groups were plotted (Figures 4(c) and 4(d)). The Log-Rank tests of Kaplan–Meier survival curves showed that the overall survival rate of the low-risk groups was significantly higher than that of high-risk groups (*p* < 0.0001). The median OS was 13 months for patients with low risk of LM and 7 months for those with high risk of LM. The median OS was 14 months for patients with low risk of BM and 7 months for those with high risk of BM. These results indicated that our nomograms could stratify the risk of LM and BM in patients with ICC and preliminarily predict survival of these patients.

### 3.5. Chemotherapy for ICC Patients with High Risk and Low Risk of LM and BM

**Figure 5:**
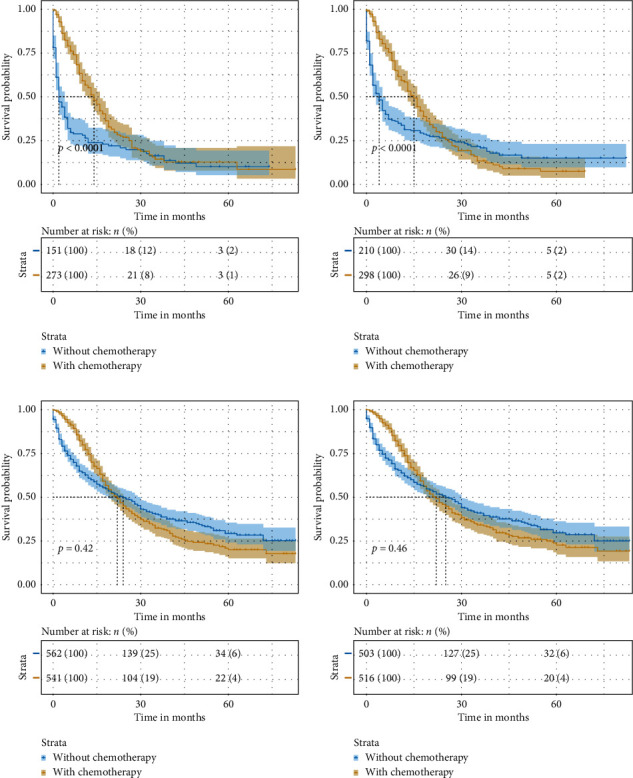
Kaplan–Meier curves of OS for ICC patients with or without chemotherapy in the (a) high-risk group of LM; (b) high-risk group of BM; (c) low-risk group of LM, and (d) low-risk group of BM. OS: overall survival; ICC: intrahepatic carcinoma; LM: lung metastasis; BM: bone metastasis.

Furthermore, Kaplan–Meier analysis was used to evaluate overall survival (OS) of patients with or without chemotherapy in high risk and low risk of LM and BM patients with ICC. The results showed that ICC patients with high risk of LM who underwent chemotherapy had better survival rates than those who did not (median OS: 9 months vs. 2 months, *p* < 0.001) (Figure 5(a)). Similarly, ICC patients with high risk of BM who underwent chemotherapy had better survival rates than those who did not (median OS: 10 months vs. 3 months, *p* < 0.001) (Figure 5(b)). In contrast, ICC patients with low risk of LM who underwent chemotherapy did not show significantly better survival rates than those who did not (median OS: 15 months vs. 11.5 months, *p* = 0.42) (Figure 5(c)). ICC patients with low risk of BM who underwent chemotherapy did not show significantly better survival rates than those who did not (median OS: 12 months vs. 15 months, *p* = 0.46) (Figure 5(d)). These results encouraged ICC patients with high risk of LM and BM to undergo chemotherapy in order to improve the prognosis of them, which indicted that our models could also be used to guide chemotherapy for ICC patients.

## 4. Discussion

It is a matter of fact that distant metastasis is a sign of advanced stage of ICC, which indicates a poor prognosis for a patient. Up to date, radically, surgical treatment has been considered the only effective therapy for ICC patients. Unfortunately, due to the hidden and nonspecific symptoms of ICC in the early stage, most patients present with advanced stages at the diagnosis time [5–7]. Nowadays, chemotherapy is the standard treatment for metastatic ICC patients, which could improve the survival of these patients to a certain extent [10]. Thus, early diagnosis of distant metastasis is very crucial for clinicians to make appropriate therapeutic strategies. The lung and bone are the two most common distant metastasis organs of ICC patients [11]. Therefore, a simple tool which can identify the risk levels of lung and bone metastases in ICC patients is extremely needed.

In the present study, by using the retrospective data from the SEER database, we established two easy-to-use nomograms based on the logistic regression model for risk visualization of LM and BM in patients with ICC. By analyzing several available variables, we could predict the risk of LM and BM in patients with ICC. Being verified by C-indexes, ROC curves, and calibration curves, the novel nomograms achieved adequate accuracy and great reliability. Furthermore, the DCAs showed that the novel nomograms had good clinical application values. In addition, internal bootstrapping validation indicated the stability of two nomograms. Using the population-based data from the SEER database made the results of our study more universal than the single-center research. To the best of our knowledge, this is the first population-based study focusing on the construction of metastatic risk prediction approaches for patients with ICC. Stratifying with cutoff point of 108 for LM and 144 for BM, we could divide ICC patients in to high-risk and low-risk groups for LM and BM, respectively. Having a higher percentage of LM or BM, two high-risk groups could benefit from chemotherapy. In general, the clinical applications of our nomograms are predicting the probability of LM and BM and selecting the optimal therapeutic strategy for ICC patients.

In view of our results, multivariate logistic analysis revealed that intrahepatic metastasis made the largest contribution to LM and BM in patients with ICC, followed by tumor size and gender. Specifically, the results showed that male patients had a higher risk of LM and BM, which was consistent with previous studies for other tumors [18, 19]. It may be caused by different hormone levels between male and female, which deserved to be confirmed by further molecular mechanism research. Several reports indicated that the tumor with larger size had the higher risk of distant metastasis [20–22]. Lymph node metastasis and higher pathological grades are important predictors for the survival of ICC patients [23]. Our study also demonstrated N stage and pathological grade of ICC patients were significantly corrected with higher risk of LM, but no significant association with these predictors was found in risk of BM. In addition, a retrospective analysis indicated that higher grade and male sex were associated with high rates of lymph node metastasis [24]. The result suggested a correlation between these risk factors.

As for clinical application of the novel nomograms, two total points of each patient with ICC were obtained by several predictors. It is easy for us to screen out the patients in high risk with LM and BM. The patients can be divided into high-risk groups and low-risk groups of LM and BM according to a total point of 108 and 144, respectively, which were calculated by the maximum value of the Youden index for the ROC curves. Patients in the high-risk groups are advised to pay close attention to the presence of LM or BM. If the probability is high, patients should be suggested to conduct more valid medical check such as high-resolution CT or PET-CT. It is important to be alert to recurrence with distant metastasis during follow-up for high-risk patients, even if metastasis is not detected during first consultation. In addition, undergoing chemotherapy might be a more appropriate treatment strategy for patients in high-risk groups. Thus, these nomograms can be used for predicting the risk of LM and BM in patients with ICC and finding out patients who need to undergo chemotherapy.

Nevertheless, there are also several limitations of the current study that should be considered. First of all, as a retrospective study, a selection bias was unavoidable. Second, the SEER database lacks the information about the specific site metastasis of the adrenal gland, kidney, and other organs, and brain metastasis data are relatively small. Thus, this study only predicted the lung and bone metastases of ICC patients. Third, the tumor biomarkers such as antigen 19, 9 (CA19, 9) may be associated with metastasis of ICC. However, we did not analyze it due to the unavailability of this information in the SEER database. In addition, the metastasis status in this study is at the time of diagnosis. Also, if we were able to identify the time of metastasis after surgery, Cox regression models could be established to predict the time when the postoperative metastases will occur, which might be better to predict the postoperative metastasis status. We will try to conduct this task in future researches. Finally, although it showed relatively good discrimination in the novel nomograms, as well as we performed bootstrapping during internal testing, further external validation at a large-scale multicenter cohort should be performed to test the applicability of the models.

In summary, despite the limitations of the current study, this is the first time to construct nomograms for predicting the risk of LM and BM in patients with ICC. These nomograms are convenient to use and have satisfactory accuracy. Specifically, larger tumor size, male, N1 stage, higher histological grade, and intrahepatic metastasis were considered as risk factors of LM in patients with ICC, while larger tumor size, male, and intrahepatic metastasis were considered as risk factors of BM in patients with ICC. By estimating the individual risk of lung and bone metastases, clinicians can conduct individualized prediction of LM and BM in patients with ICC and give more favorable treatment recommendations, such as undergoing chemotherapy.

## Figures and Tables

**Table 1:** Demographic and clinical characteristics of intrahepatic cholangiocarcinoma patients.

Variables	With LM (82)	Without LM (1445)	With BM (70)	Without BM (1457)
Age				
<45	3 (3.7)	76 (5.3)	4 (5.7)	75 (5.1)
45–65	41 (50.0)	575 (39.8)	34 (48.6)	582 (39.9)
≥65	38 (46.3)	794 (54.9)	32 (45.7)	800 (54.9)
Gender				
Female	26 (31.7)	714 (49.4)	18 (25.7)	722 (49.6)
Male	56 (68.3)	731 (50.6)	52 (74.3)	735 (50.4)
Race				
White	71 (86.6)	1149 (79.5)	61 (87.1)	1159 (79.5)
Black	3 (3.7)	118 (8.2)	2 (2.9)	119 (8.2)
Other	8 (9.8)	178 (12.3)	7 (10.0)	179 (12.3)
Histological grade				
Well/moderate	34 (41.5)	868 (60.1)	18 (25.7)	722 (49.6)
Poor/undifferentiated	48 (58.5)	577 (39.9)	52 (74.3)	735 (50.4)
*T* stage				
*T*1	16 (19.5)	508 (35.2)	23 (32.9)	501 (34.4)
*T*2	43 (52.4)	643 (44.5)	33 (47.1)	653 (44.8)
*T*3	19 (23.2)	186 (12.9)	12 (17.1)	193 (13.2)
*T*4	4 (4.9)	108 (7.5)	2 (2.9)	110 (7.5)
Marital status				
Single	26 (31.7)	510 (35.3)	17 (24.3)	519 (35.6)
Married	56 (68.3)	935 (64.7)	53 (75.7)	938 (64.4)
*N* stage				
*N*_0_	42 (51.2)	1048 (72.5)	43 (61.4)	1047 (71.9)
*N*_1_	40 (48.8)	397 (27.5)	27 (38.6)	410 (28.1)
Size				
≤5 cm	10 (12.2)	579 (40.1)	10 (14.3)	579 (39.7)
5–10 cm	39 (47.6)	649 (44.9)	40 (57.1)	648 (44.5)
>10 cm	33 (40.2)	217 (15.0)	20 (28.6)	230 (15.8)
Intrahepatic metastasis				
No	56 (68.3)	1390 (96.2)	55 (78.6)	1391 (95.5)
Yes	26 (31.7)	55 (3.8)	15 (21.4)	66 (4.5)

LM: lung metastasis, BM: bone metastasis, *n* (%) for categorical variables.

**Table 2:** Univariate and multivariate logistic regressions of predictors of lung metastasis from ICC patients.

Variables	Univariate logistic		Multivariable logistic	
	OR (95% CI)	*p* value	OR (95% CI)	*p* value
Age				
<45	1 (reference)			
45–65	1.806 (0.637–7.587)	0.333		
≥65	1.212 (0.426–5.100)	0.753		
Gender				
Female	1 (reference)		1 (reference)	
Male	2.101 (1.320–3.438)	0.002	2.253 (1.365–3.817)	0.002
Race				
White	1 (reference)			
Black	0.411 (0.100–1.127)	0.137		
Other	0.727 (0.318–1.449)	0.404		
Histological grade				
Well/moderate	1 (reference)		1 (reference)	
Poor/undifferentiated	2.124 (1.357–3.360)	0.001	1.791 (1.094–2.957)	0.021
*T* stage				
*T*1	1 (reference)		1 (reference)	
*T*2	2.123 (1.206–3.924)	0.012	1.152 (0.618–2.226)	0.664
*T*3	3.243 (1.634–6.516)	<0.001	1.531 (0.713–3.285)	0.271
*T*4	1.176 (0.332–3.280)	0.420	0.598 (0.159–1.802)	0.397
Marital status				
Single	1 (reference)			
Married	1.174 (0.736–1.922)	0.508		
*N* stage				
*N*_0_	1 (reference)		1 (reference)	
*N*_1_	2.514 (1.603–3.939)	<0.001	1.611 (0.978–2.641)	0.050
Size				
≤5 cm	1 (reference)		1 (reference)	
5–10 cm	3.479 (1.790–7.428)	<0.001	3.171 (1.565–7.014)	0.002
>10 cm	8.805 (4.421–19.123)	<0.001	8.133 (3.867–18.563)	<0.001
Intrahepatic metastasis				
No	1 (reference)		1 (reference)	
Yes	11.734 (6.793–19.980)	<0.001	10.431 (5.768–18.707)	<0.001

ICC: intrahepatic carcinoma, OR: odds ratio, CI: confidence interval.

**Table 3:** Univariate and multivariate logistic regressions of predictors of bone metastasis from ICC patients.

Variables	Univariate logistic		Multivariable logistic	
	OR (95% CI)	*p* value	OR (95% CI)	*p* value
Age				
<45	1 (reference)			
45–65	1.095 (0.422–3.744)	0.867		
≥65	0.750 (0.288–2.568)	0.597		
Gender				
Female	1 (reference)		1 (reference)	
Male	2.838 (1.676–5.027)	<0.001	2.870 (1.663–5.176)	<0.001
Race				
White	1 (reference)			
Black	0.319 (0.052–1.039)	0.115		
Other	0.743 (0.305–1.543)	0.466		
Histological grade				
Well/moderate	1 (reference)			
Poor/undifferentiated	1.303 (0.803–2.108)	0.280		
*T* stage				
*T*1	1 (reference)			
*T*2	1.101 (0.642–1.920)	0.730		
*T*3	1.354 (0.641–2.727)	0.407		
*T*4	0.396 (0.063–1.365)	0.214		
Marital status				
Single	1 (reference)		1 (reference)	
Married	1.725 (1.010–3.097)	0.055	1.331 (0.7628–2.433)	0.332
*N* stage				
*N*_0_	1 (reference)		1 (reference)	
*N*_1_	1.603 (0.967–2.613)	0.061	1.166 (0.685–1.946)	0.562
Size				
≤5 cm	1 (reference)		1 (reference)	
5–10 cm	3.574 (1.843–7.620)	<0.001	3.601 (1.834–7.751)	<0.001
>10 cm	5.035 (2.373–11.371)	<0.001	4.551 (2.107–10.434)	<0.001
Intrahepatic metastasis				
No	1 (reference)		1 (reference)	
Yes	5.748 (2.997–10.485)	<0.001	5.198 (2.632–9.817)	<0.001

ICC: intrahepatic carcinoma, OR: odds ratio, CI: confidence interval.

## Data Availability

The data availability was revised as The datasets analyzed during the current study are available in the SEER repository and can be obtained from: (https://seer.cancer.gov.https://seer.cancer.gov.).

## Abbreviations

ICC: Intrahepatic cholangiocarcinoma
LM: Lung metastasis
BM: Bone metastasis
SEER: Surveillance, Epidemiology, and End Results
HCC: Hepatocellular carcinoma
PET-CT: Positron emission tomography-computed tomography
OR: Odds ratios
ROC: Receiver operating characteristic
DCA: Decision curve analyses
AJCC: American Joint Committee on *Cancer*
OS: Overall survival.
